# Curcumin sensitizes response to cytarabine in acute myeloid leukemia by regulating intestinal microbiota

**DOI:** 10.1007/s00280-021-04385-0

**Published:** 2022-01-23

**Authors:** Junmin Liu, Wei Luo, Qiuru Chen, Xing Chen, Gang Zhou, Hongbo Sun

**Affiliations:** Department of Hematology, Affiliated Hospital of Southern Medical University, Shenzhen Longhua District People’s Hospital, No. 38 Jinglong Jianshe Road, Longhua District, Guangdong Province 518109 Shenzhen, People’s Republic of China

**Keywords:** Curcumin, Cytarabine, Acute myeloid leukemia, Microbiota, Cholesterol, SQLE

## Abstract

**Purpose:**

To address whether Curcumin has synergistic effect with cytarabine (Ara-C) in treating acute myeloid leukemia (AML).

**Methods:**

A xenograft AML mouse model was established by injecting HL-60 cells into tail vein of mice to assess the function of Curcumin. Mononuclear cells (MNCs) isolated from AML mice and AML cell lines were used to examine the effect of Curcumin. Metagenomics and metabolomics were used to evaluate the alteration of intestinal microbiota and the change of metabolites in MNCs.

**Results:**

Curcumin treatment sensitized response to Ara-C in MNCs of AML mice, but had no direct effect on AML cell lines. Metagenomics revealed an alteration of intestinal microbiota with Curcumin treatment, which contributes to sensitized response to Ara-C. Curcumin treatment led to enhanced intestinal intact to sensitize response to Ara-C in AML mice, through reducing mucus degrading bacteria. Metabolomics demonstrated that Curcumin treatment led to decreased cholesterol in MNCs of AML mice. Further study proved that Curcumin treatment resulted in inhibition of SQLE, a key enzyme of cholesterol biosynthesis, to increase sensitivity to Ara-C.

**Conclusion:**

Curcumin sensitizes response to Ara-C through regulating microbiota, highlighting the importance of intestinal intact strengthening in chemoresistant therapy. Moreover, aiming at cholesterol synthesis is promising in AML treatment.

**Supplementary Information:**

The online version contains supplementary material available at 10.1007/s00280-021-04385-0.

## Introduction

Acute myeloid leukemia (AML) originates from the differentiation block of myeloid cells in the bone marrow (BM) [1]. As a malignant tumor of the blood system, the survival rate of AML is low (< 40%), which is mainly due to chemoresistance [2]. Despite a high rate of complete remission after treatment with chemotherapy drugs, the relapse rate remains very high and the prognosis very poor [1]. Therefore, ameliorating chemoresistance remains to be a clinical challenge.

Curcumin is a natural phenolic compound extracted from curcuma longa, which exerts a wide range of biological effects, such as anti-tumor, anti-inflammatory, anti-oxidation and anti-fibrosis [3, 4]. Moreover, Curcumin was also reported to affect energy metabolism to increase energy expenditure as well as improve insulin sensitivity in obese mice [5, 6]. Energy metabolism also plays crucial roles in response to chemotherapy. Several studies have reported that targeting energy metabolism could sensitize resistant cells to chemotherapy, such as oxidative phosphorylation inhibitor redirected metabolism toward glycolysis to sensitize resistant cells to cytarabine (Ara-C) in AML [7]. On the other hand, Curcumin could affect intestinal microbiota to prevent renal failure [6]. Conversely, intestinal microbiota could also enhance the effect of Curcumin in ameliorating HFD-induced obesity by enhancing Ucp1-dependent thermogenesis through regulating bile acids metabolism [8], suggesting the interaction between intestinal microbiota and Curcumin in curing diseases.

In the present study, we will investigate the synergistic effect between Curcumin and Ara-C using AML mouse xenograft mouse model and its potential mechanism.

## Materials and methods

### Xenotransplantation of human leukemic cells

Xenograft AML model was established using a regular method as previously described [9, 10]. Briefly, Cultured HL-60 cell line was washed twice in phosphate-buffered saline (PBS) and cleared aggregates and debris using a 0.2-mm cell filter, which were suspended in PBS at a final concentration of 2 million cells per 200 μl of PBS per mouse for intravenous injection. As the molecular changes is different between male and female patients, we used male mice for the model establishment. Xenograft tumors were generated by injecting AML cells (in 200 μl of PBS) in the tail vein of 6-week-old male NSG mice. Daily monitoring of mice for symptoms of disease (ruffled coat, hunched back, weakness and reduced motility) determined the time of killing for injected animals with signs of distress. Mice were raised in the animal room of Shnezhen Longhua New District People's Hospital. Germ free mice were obtained from Cyagen and raised in Germ free package. The committee of Shnezhen Longhua New District People's Hospital approved this study. All animal experiments comply with the ARRIVE guidelines and carried out in accordance with the National Institutes of Health guide for the care and use of Laboratory animals (NIH Publications No. 8023, revised 1978).

### Cytarabine, curcumin, VSL#3 and terbinafine treatment

Following the mice engraftment (symptoms of disease at week 3 after cell injection), we started to treat them with daily intraperitoneal injections of 30 mg/kg Ara-C for 5 days [10]. The in vivo experiments were performed in NSG recipients transplanted. For negative control, NSG mice were treated daily with PBS for 5 days. Mice were monitored for toxicity and provided with nutritional supplements as needed. Curcumin was proved to be non-toxic and it was administrated to mice (200 mg/kg body weight daily) intraperitoneally when Ara-C was treated. VSL#3 (15 mg, mixture of Lactobacilli, Bifidobacteria and Streptococcus) was orally administrated to mice daily one week before Ara-C was treated. Terbinafine was also orally administrated to mice (80 mg/kg body weight daily) when Ara-C was treated.

### MNCs isolation and cell culture

MNCs were isolated from mice with a standard Ficoll–Hypaque gradient separation method as previously used [11]. The committee of Shenzhen Longhua New District People’s Hospital approves this experiment. MNCs HL-60 and THP-1 (obtained from ATCC) were cultured in RPMI-1640 medium (Gibco; Thermo Fisher Scientific, Inc.) supplemented with 10% FBS, 100 U/mL penicillin, and 100 mg/mL streptomycin at 37 °C in a humidified atmosphere containing 5% CO2.

## Western blotting

Protein was extracted from cells using cold RIPA buffer with protease inhibitors and phosphatase inhibitor. Then, protein was separated in 10% SDS-PAGE and transferred into nitrocellulose membrane (NC). After blocking for 30 min with 1% BSA, the membranes were incubated with primary antibodies at 4 °C overnight. Then, membrane was incubated with secondary antibodies for 1 h at room temperature. Finally, protein was detected using ChemiDoc MP Imaging System.

### Intestine permeability assay

To determine intestinal permeability, mice were starved overnight, followed by gavaging FITC-dextran (Sigma #FD4) orally (44 mg/100 g body weight). After 4 h, mice were anesthetized, then blood was collected by cardiac puncture using 1 ml syringe and finally mice were killed. Serum was separated using Tubes containing coagulant (BD #365,968) and diluted with an equal volume of PBS. 100 μl of diluted serum was added to a 96-well microplate [12]. The concentration of FITC in serum was determined by spectrophotofluorometry (BioTek).

### SQLE over-expression

pRL-cyto-megalovirus (pCMV)–SQLE (RC202008) and pCMV-entry control plasmids (OriGene) were transfected into cells using Lipofectamine 2000 (Invitrogen) according to the manufacturer’s instructions.

### Cell viability and apoptosis assay

AML cells were seeded into 96-well plates (500 cells/well). Then, cells were incubated with drugs [(Ara-C HL-60:0.1 µM; THP-3 µM), Curcumin (serial concentrations)]. CCK-8 reagent (10 μL) were added to 96-well plates, incubating for another 1 h at 37 °C in dark. The absorbance was studied at an OD of 450 nm by a microplate reader. Cell proliferation rate (%) = OD value of experimental well/ OD value of blank well × 100%. Apoptosis was evaluated using an annexin–phycoerythrin/7–aminoactinomycin D staining kit (BD Biosciences).

### Cholesterol concentration

MNCs (106) were harvested, and cholesterol concentration was assessed by Cholesterol Quantification kit (ab65359, Abcam) according to the manufacturer’s instruction.

### Shot-gun metagenome sequencing and analyses

Stool was collected from Cur/A- and Ara-C-treated AML mice. DNA of stool was extracted using PowerFecal DNA isolation kit (QIAGEN) according to the manufacturer’s instruction. DNA concentration was measured using NanoDrop. Amplified library was generated using Era XT adapters. HiSeq Illuminex 2500 was used for sequencing, which was performed to obtain DNA (125 bp) paired end reads to a depth of 10 G base pairs per sample. For sequencing data, BWA and SAM tools were used for host DNA removal and quality control as previously described [13, 14]. Then, Kraken 2 and Bracken were applied for species-level taxonomic profiling using high-quality reads [16]. Bacteria species with adjusted *p*-value < 0.05 and |Log2(fold change)|> 1 were considered statistically significant.

### Metabolomics profiling and analyses

Metabolomics was performed in the company of Novogene. Briefly, MNCs (106) was homogenized on ice and dissolved in 500 uL of cold water. Then, samples were vortexed and centrifuged (10,000*g*) for 15 min. Supernatant was collected and the pellets were further extracted using 500 uL of cold methanol. Cells’ extracts were mixed and centrifuged with 10,000*g* for 15 min. Supernatant was used for LC–MS analysis. A waters Acquity Ultra-high-Performance LC system, equipped with a BEHC18 column (Milford, USA), was applied using chromatographic analysis. The mobile phases consisted of solvents A and B. The elution gradient program for MNCs was: 0–1% B for 0.5 min; 1–30% B from 0.5 to 5 min; 30–50% B from 5 to 13 min and 50–100% B from 13 to 17 min; the flow rate was 0.45 ml/min. Mass spectra were obtained from a Waters SYNAPT G2 HDMS (Waters Corp., Manchester, UK) TOF mass spectrometer combined with an ESI source with positive ion scan mode.

The metabolomic data were analyzed using Metabo AnalystR. Wilcoxon rank-sum test was used to determined significantly altered metabolites. Metabolites with adjusted *p*-value < 0.05 and |Log2 (fold change)|> 1 were considered significant change. The heatmap was plotted using R package Complex Heatmap.

### DNA extraction and qPCR of microbiota in mouse stool

Total DNA was extracted from aliquots of 100 mg of mouse stool. Markedly altered bacteria were evaluated with quantitative PCR (qPCR) analyses, as previously described [16].

### Statistical analysis

All statistical tests were performed using SPSS or GraphPad Software. Data are presented as mean ± SD. Cell viability assay was analyzed with repeated-measures ANOVA. Comparison between two groups was analyzed with Paired two-tailed Student’s *t* tests.

## Results

### Curcumin sensitizes response to Ara-C in xenograft model but not in AML cells

**Fig. 1:**
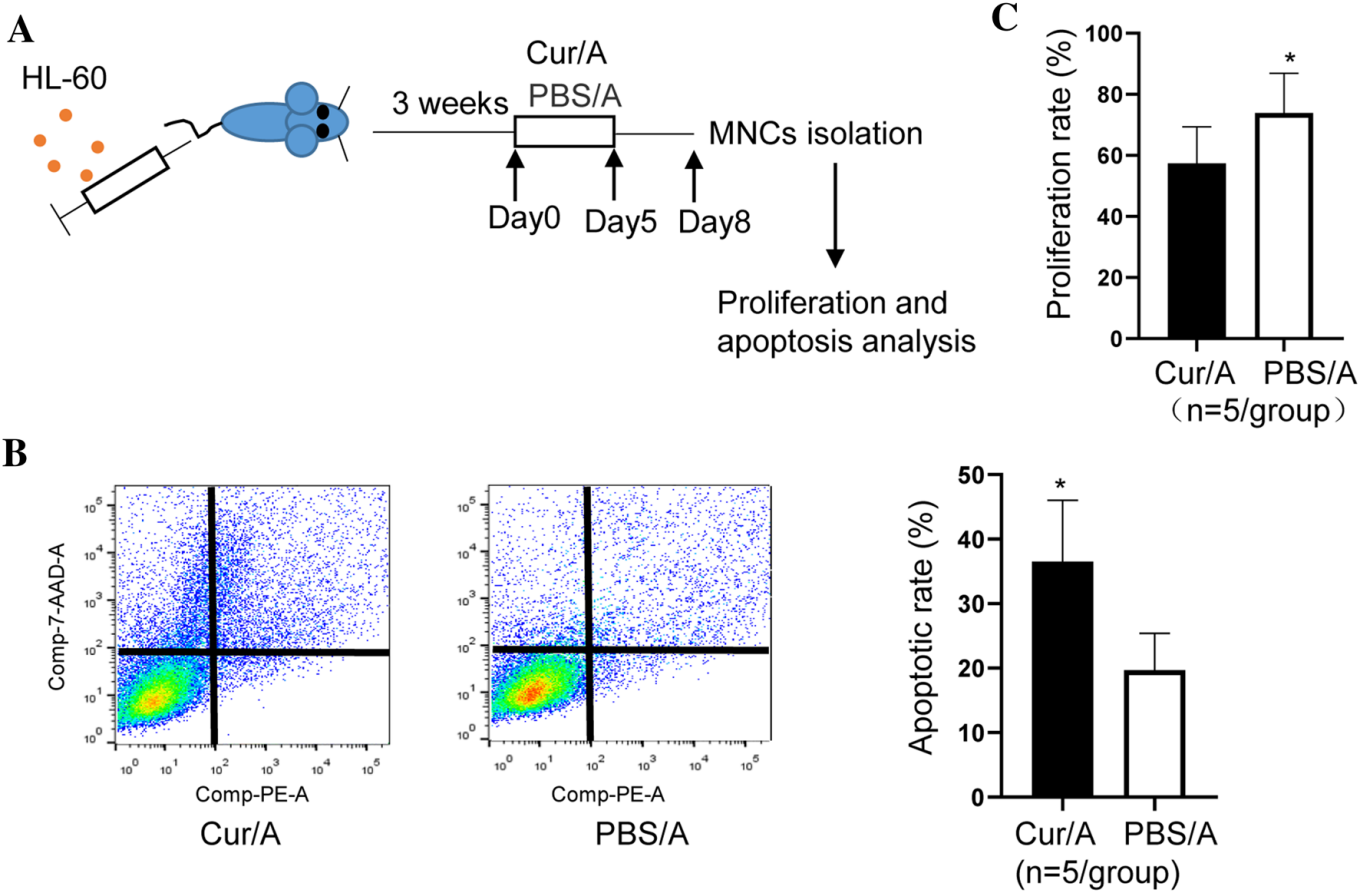
Curcumin and Ara-C displayed synergistic effect in mice but not in cell lines. **A** Establishment of AML mouse model and experimental design. **B**, **C** MNCs isolated from Cur/A AML mice showed significantly increased apoptosis in concomitant with decreased cell proliferation. *p < 0.05

To address the synergistic effect between Curcumin and Ara-C, HL-60 cells were xenografted into male NSG-immunodeficient mice by tail vein as previously described. At week 3 after HL-60 cells injection, the symptoms of disease appeared. After the establishment of disease, mice were treated with either Curcumin + Ara-C (Cur/A) or PBS + Ara-C (PBS/A) for 5 days consecutively. BM samples were collected on day 8 and monoclonal cells (MNCs) were isolated (Fig. 1A). CCK-8 assay showed that MNCs isolated from Cur/A group displayed significantly reduced cell proliferation rate compared to PBS/A group (Fig. 1B). Apoptosis assay also revealed that Cur/A led to markedly increase of MNCs compared to the PBS/A group (Fig. 1C). To further confirm this observation, we performed in vitro assay that HL-60 and THP-1 cells were treated with serial concentrations of Curcumin (100, 50, 25, 12, 6, 3, 1 and 0 μM) and Ara-C (3 μM) (Cur/A or PBS/A treatment). Inconsistent with what observed in the animal model, we did not see significant alteration in Cur/Ara co-treated group compared to PBS/A-treated group as evidenced by CCK-8 and apoptosis assay (Fig S1A–D).

### Curcumin leads to altered intestinal microbiota

**Fig. 2:**
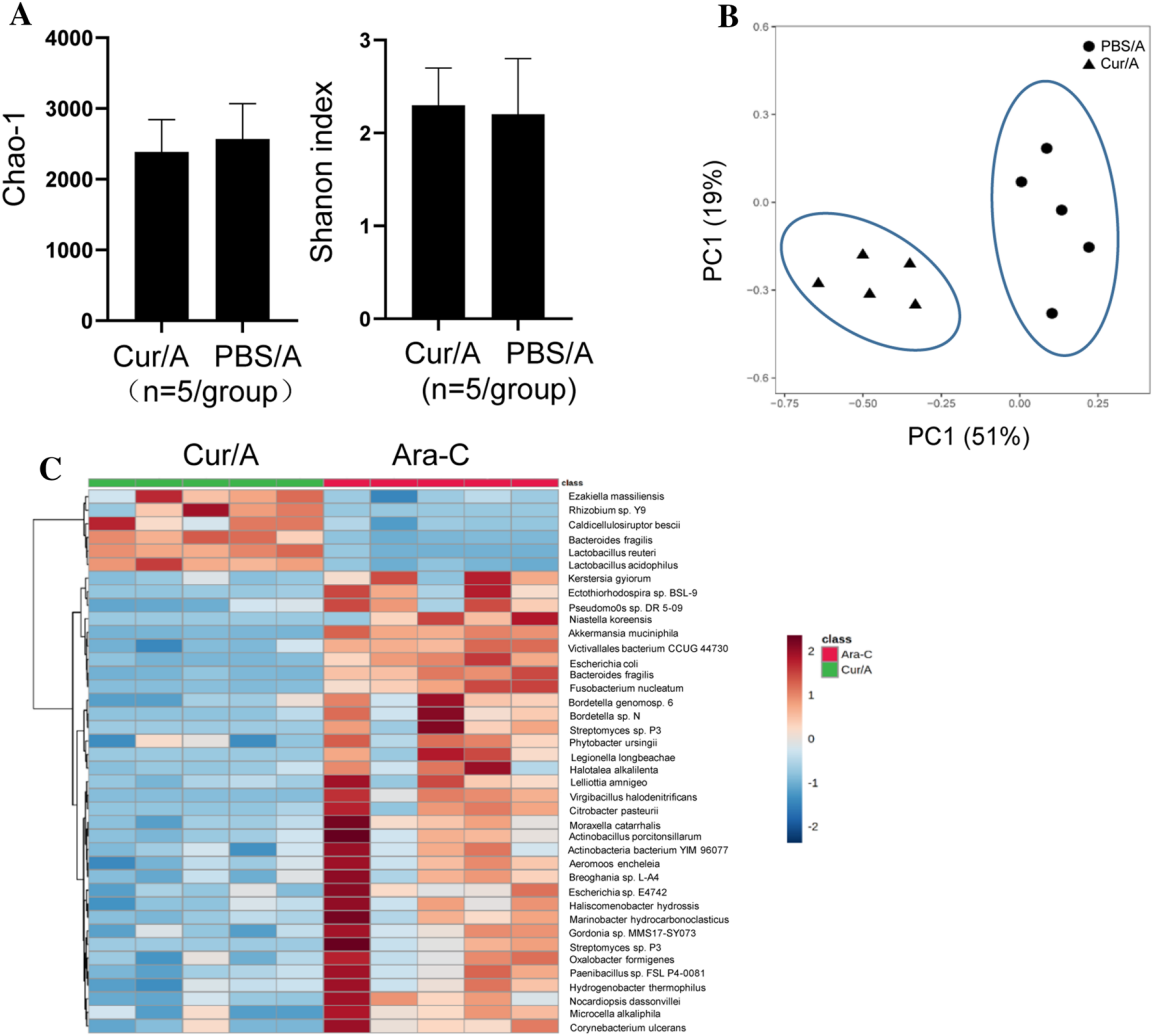
Curcumin treatment led to intestinal microbiota alteration. **A** α-diversity showed no significant change of bacteria diversity alteration with curcumin treatment as evidenced by Chao-1 and Shannon index analysis. **B** PCA assay revealed that Curcumin treatment resulted in markedly altered microbiota composition. **C** Heatmap showed that Curcumin induced probiotics enrichment while pathogenic bacteria were reduced

Due to the inconsistence between the in vivo and in vitro analysis, we asked whether this inconsistence is associated with intestinal microbiota which have been established relationship with AML [17]. To address this question, we first compared the microbiota profiling between Cur/A-treated mice and PBS/A-treated mice. α-diversity analysis revealed an unchanged enrichment of bacteria species as determined by Shannon index and Chao-1 (Fig. 2A). However, principal component analysis (PCA) based on species-level revealed a significant alteration of intestinal microbiota composition between Cur/A and PBS/A group (Fig. 2B). Heatmap showed that *Lactobacillus Acidophilus* (*L. acidophilus*), *Bifidobacterium bifidum* (*B. bifidum*) and *Lactobacillus reuteri* (*L. reuteri*) were significantly increased while pathologic bacteria, including *Bacteroides fragilis* (*B. fragilis*), *Escherichia coli* (*E. Coli*), *Fusobacterium nucleatum* (*Fn*) and *Akkermansia muciniphila* (*A. muciniphila*) were significantly reduced (Fig. 2C and Table S1). These observations indicate that Curcumin combined with Ara-C is associated with a different microbiota composition compared to Ara-C alone.

### Altered intestinal microbiota is involved in Curcumin-mediated increased response to Ara-C

**Fig. 3:**
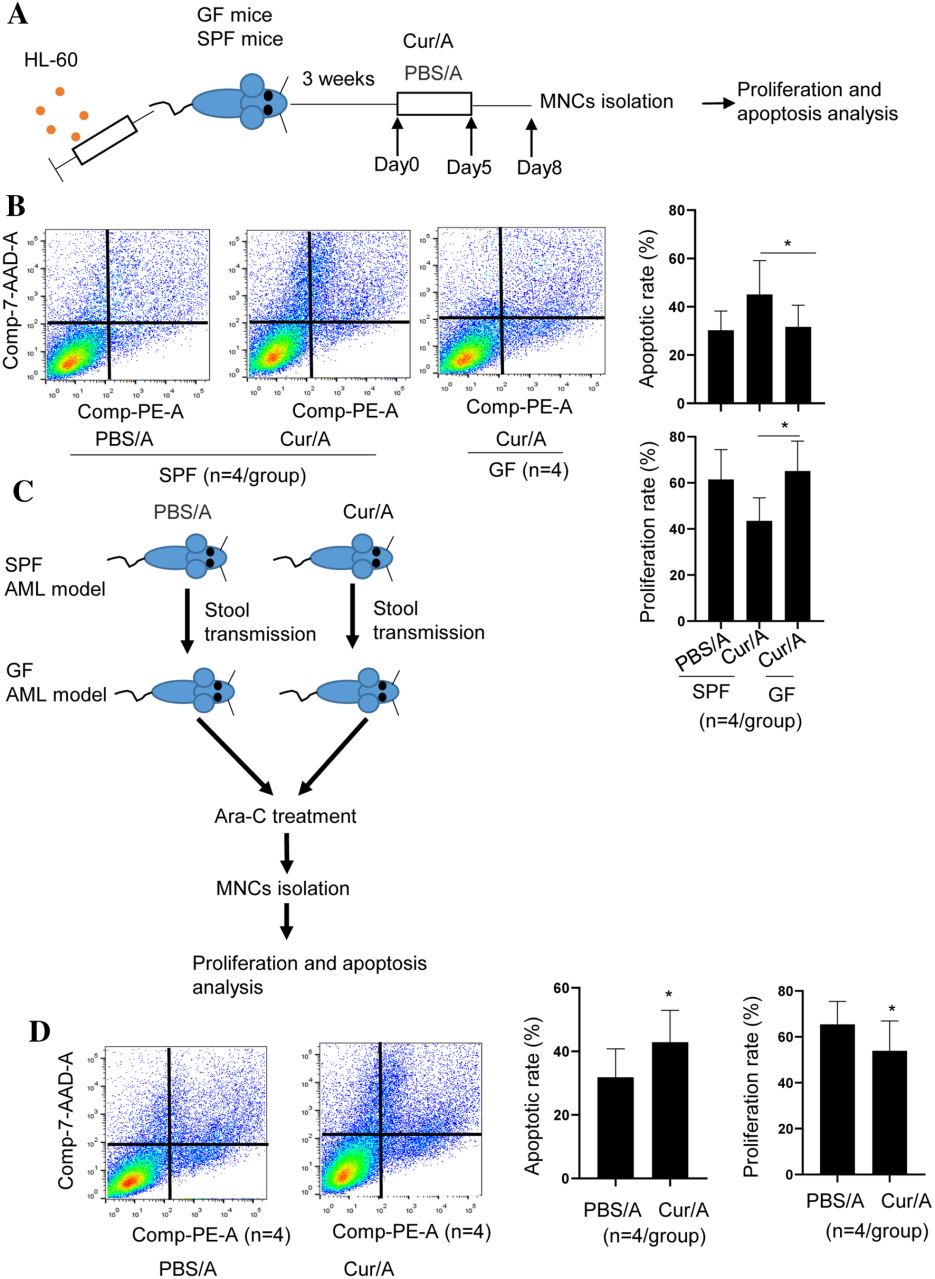
Curcumin-induced microbiota alteration sensitized response to Ara-c. **A** Establishment of AML mouse model in SPF and GF mice. **B** Apoptosis and proliferation assay demonstrated that Curcumin had no effect on sensitizing response to Ara-c under GF condition. **C** Experimental design for stool transplantation. **D** Apoptosis and proliferation assay revealed that stool of Cur/A-treated mice sensitized response to Ara-C. *p < 0.05

To determine whether altered microbiota induced by Curcumin is associated with sensitized response to Ara-C, we used germ free mice (GF) to develop AML xenograft model, followed by Curcumin and Ara-C treatment as mentioned above. Then MNCs were isolated for viability and apoptosis assay (Fig. 3A). The time of the appearance of disease symptoms in GF mice was similar with the SPF mice. Interestingly, the synergistic effect between Curcumin and Ara-C disappeared in GF mice (Fig. 3B), suggesting Curcumin regulate microbiota to enhance response to Ara-C in AML. To further confirm this observation, GF mice were treated with stools of Cru/A-treated mice or stools of PBS/A mice (Fig. 3C). To confirm the colonization of bacteria, we checked the volume of altered bacteria using qPCR. It showed that *B.fragilis, E. Coli and Fn* were significantly reduced in Cur/A-treated-mice-stool-gavaged mice in concomitant with decreased *B. fragilis, E. Coli, Fn and A.muciniphila*, indicating a successful colonization (Fig. S2). Therefore, we treated the mice with Ara-C. Consistently, Cur/A-treated-mice-stool-gavaged mice displayed significantly reduced viability in concomitant with increased apoptosis in MNCs (Fig. 3D). These results indicate that Curcumin regulates intestinal microbiota to sensitize response to Ara-C.

### Curcumin enhances intestinal intact to sensitize response to AML

**Fig. 4:**
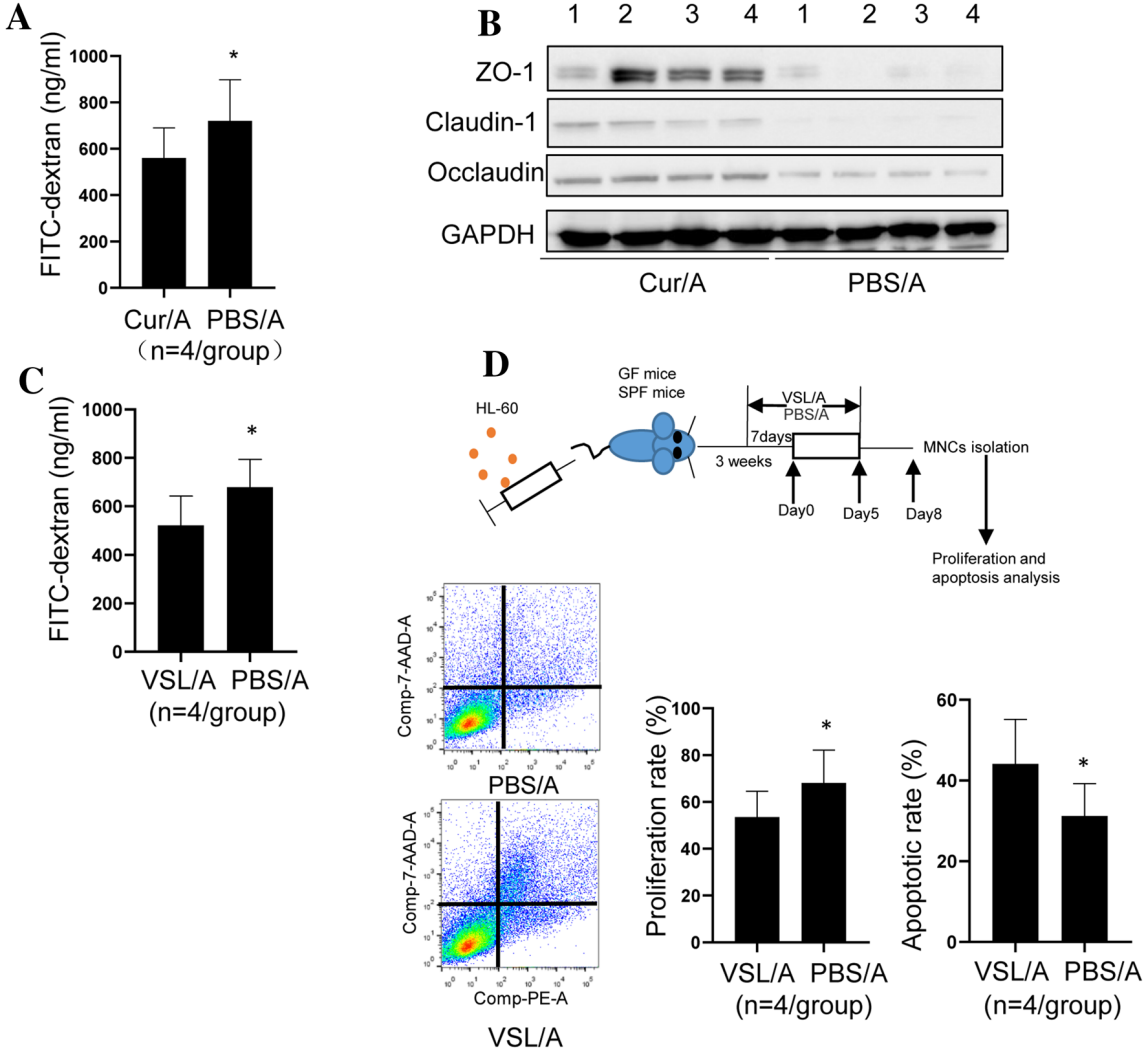
Curcumin treatment enhanced intestinal intact. **A** Fitc-Dextran assay showed that Curcumin treatment led to decreased intestinal permeability. **B** TJPs, ZO-1, Occludin and Claudin-1, were up-regulated in Cur/A-treated mice. **C** Probiotics treatment reduced intestinal permeability compared to control group. **D** Apoptosis and proliferation assay showed significantly increased apoptosis and decreased proliferation rate in MNCs of probiotics-treated AML mice. *p < 0.05

Intestinal intact is important in separating intestinal pathogen from epithelial cells to avoid pathogen invasion [18]. Mucus and tight junction proteins (TJPs) are key component to maintain intestinal intact [19]. As we observed the reduction of bacteria with the function of degrading intestinal mucus in Curcumin-treated mice, we asked whether Curcumin could strengthen intestinal intact. To address this question, FITC-dextran was administrated to mice 4 h before killing. Then, blood was collected for FITC determination. Consistent with our hypothesis, the concentration of FITC-dextran was significantly reduced in serum of Cur/A-treated mice (Fig. 4A). Tight junction proteins ZO-1, occludin and claudin-1 were also increased in Cur/A-treated mice (Fig. 4B). Next, we investigated whether enhanced intestinal intact could sensitize response to Ara-C in AML. Probiotics (VSL#3) was previously reported to strengthen intestinal intact. AML mice were treated with VSL#3 (15 mg in 200ul PBS per mouse and treated to mice one week before Ara-c treatment) plus Ara-C (VSL/A) or PBS/A (Fig. 4C). It turned out that MNCs of VSL/A-treated AML mice showed significantly decreased proliferation rate and increased apoptosis compared to PBS/A-treated AML mice (Fig. 4D). These results indicate that Curcumin enhances response to Ara-C by strengthening intestinal intact.

### Curcumin-induced microbiota alteration affects intracellular cholesterol synthesis

**Fig. 5:**
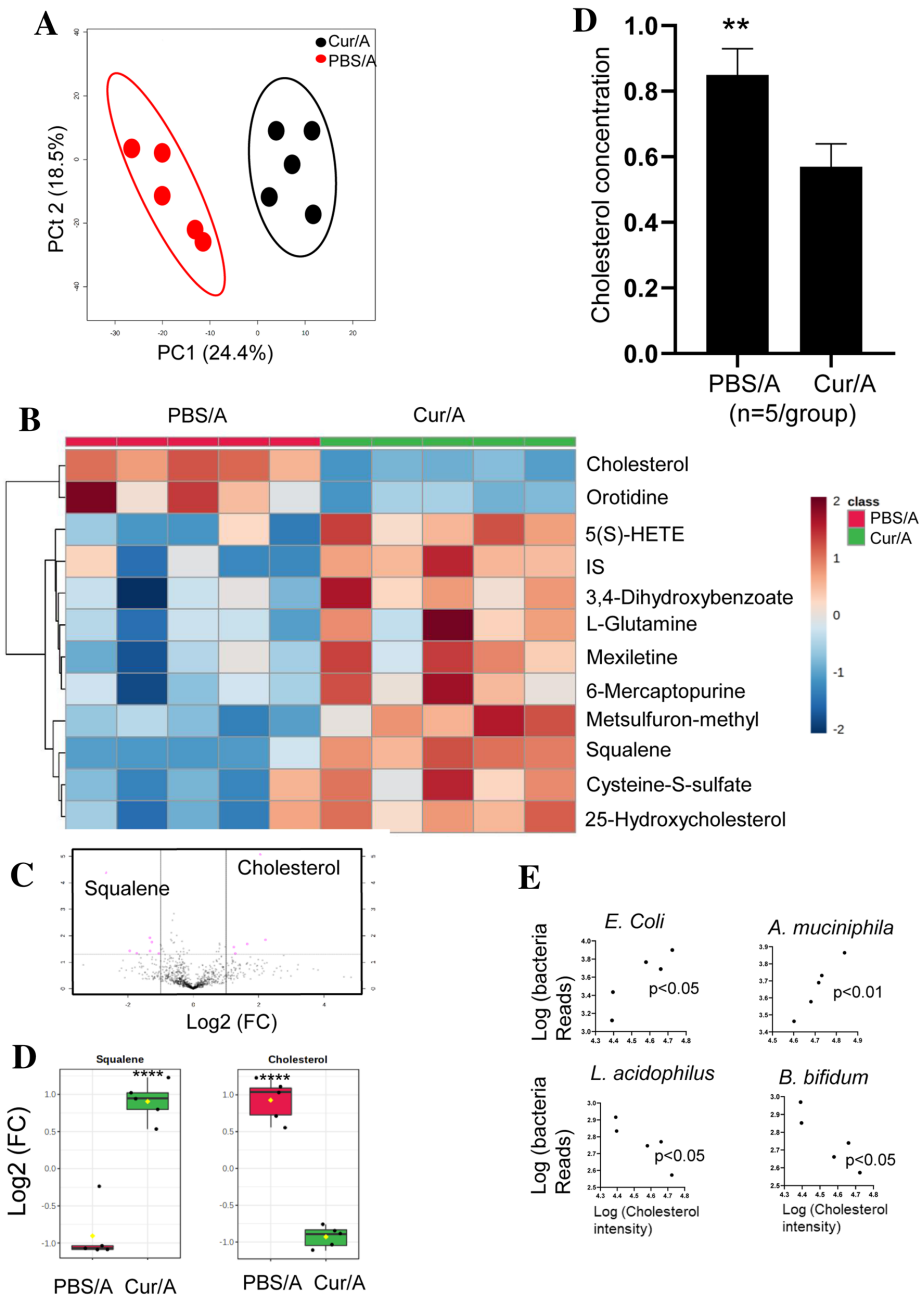
Curcumin-induced microbiota alteration led to change of metabolites in MNCs of AML mice. **A** PCA analysis saw an altered metabolomics of MNCs between Cur/A- and PBS/A-treated AML mice. **B** Heatmap showed markedly altered metabolites. **C** Volcano plot revealed that cholesterol and squalene were located at the outlier of significantly changed metabolites. **D** Curcumin treatment led to significantly down-regulated cholesterol in MNCs of AML mice. **p < 0.01, ****p < 0.0001

Cellular metabolites were proved to be associated with chemoresistance [20]. To disclose the mechanism of Curcumin sensitizing response to Ara-C, we performed metabolomics for BM MNCs collected from Cur/A- and PBS/A-treated mice. The composition of metabolites in MNCs of the two groups of AML mice was significantly different as determined by principal composition analysis (PCA) (Fig. 5A). Heatmap showed that in Cur/A-treated MNCs, some metabolites were decreased while others were increased (Fig. 5B and Table S2). Of note, cholesterol, which was enriched in PBS/A-treated MNCs, was located at the outlier of volcano plot, whereas squalene, which was enriched in Cur/A-treated MNCs, was located at the outlier of volcano plot (Fig. 5C). Next, we further examined the cholesterol level directly in the MNCs. Consistently, cholesterol level was significantly reduced in Cur/A-treated MNCs compared to PBS/A-treated MNCs (Fig. 5D).Correlation analysis was performed to determine the potential association between altered gut microbes and cholesterol of PBS/A-treated mice. It showed that *A. muciniphila and E.Coli* were positively correlated with cholesterol, whereas *L. acidophilus* and *B. bifidum* were negatively correlated with cholesterol (Fig. 5E). These observations indicate that Curcumin-induced microbiota alteration reduced intracellular cholesterol level of MNCs.

### Curcumin-induced microbiota sensitizes response to Ara-C by suppressing SQLE

**Fig. 6:**
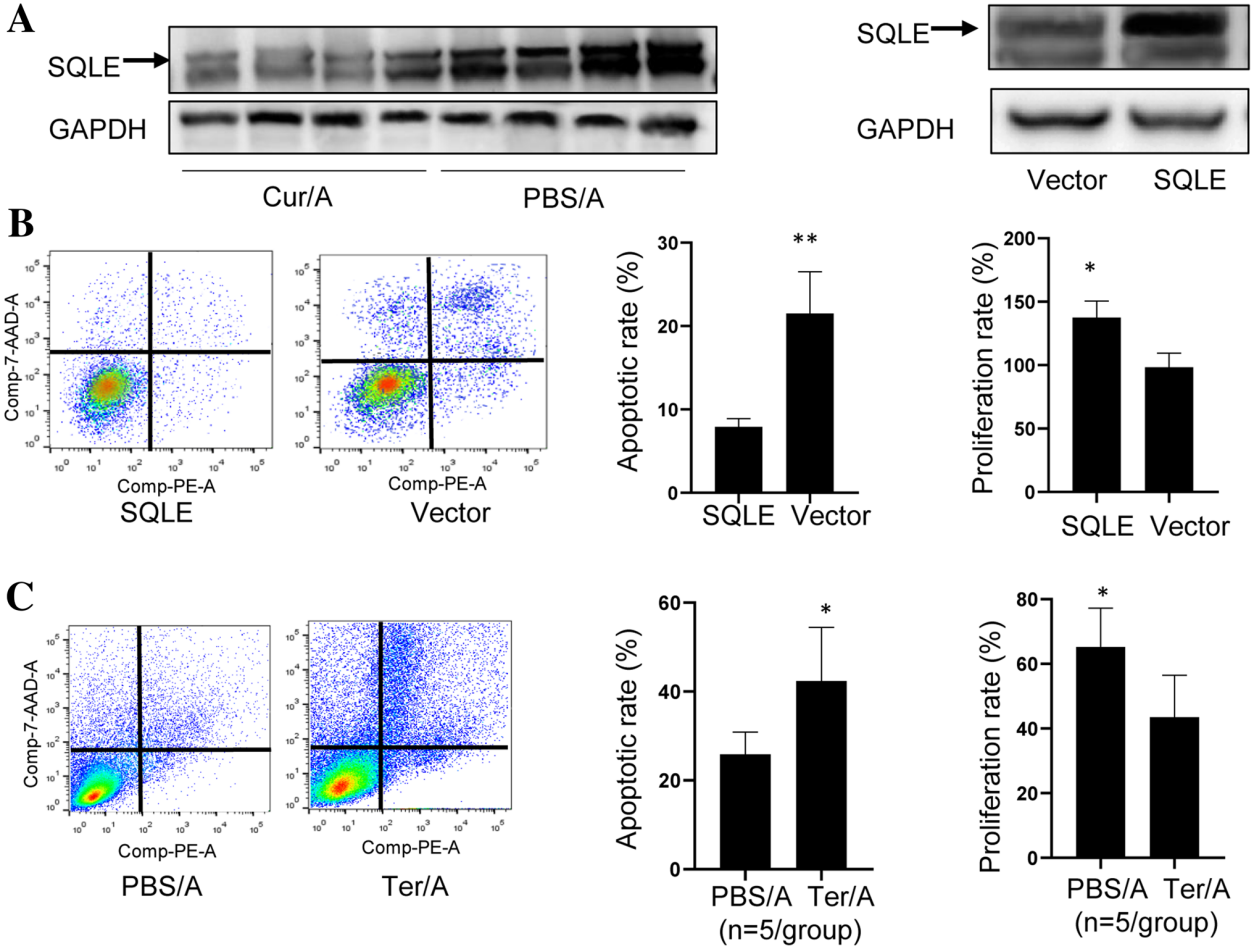
Curcumin sensitized response to Ara-C through suppressing SQLE. **A** SQLE was up-regulated in MNCs of Cur/A-treated AML mice. **B** SQLE over-expression in THP-1 cells led to markedly decreased apoptosis as well as increased proliferation. **C** SQLE inhibitor resulted in significantly increased apoptosis and reduced proliferation in MNCs of AML mice. *p < 0.05, **p < 0.01

It was reported that cholesterol accumulation was associated with chemoresistance [21]. On the other hand, we noticed that Squalene acts as the substrate of Squalene Epoxidase (SQLE) for cholesterol biosynthesis. So, we hypothesized that SQLE is involved in the Curcumin-mediated sensitized response to Ara-C in AML. WB showed that SQLE expression was inhibited in MNCs of Cur/A-treated AML mice compared to MNCs of PBS/A-treated mice (Fig. 6A) To further confirm the function of SQLE, THP-1 cells were transfected with SQLE plasmids. As a result, SQLE over-expression led to a significant reduction of cell apoptosis as well as increased cell viability (Fig. 6B). To further assess the function of SQLE we used Terbinafine, a selective SQLE inhibitor, to treat AML mice. Similar with what observed in cells, SQLE inhibition resulted in sensitized response to Ara-C in MNCs of AML mice (Fig. 6C). These results suggest that Curcumin sensitizes response to Ara-C in AML by suppressing SQLE.

## Discussion

In the present study, we investigated the effect of Curcumin on the response to Ara-C. A xenograft AML mouse model was established to assess the synergistic effect between Curcumin and Ara-C. We found that Curcumin, unlike the observations in other disease, could not directly sensitize response to Ara-C as we did not see a synergistic effect between Curcumin and Ara-C in the in vitro analysis. Instead, it regulates intestinal microbiota to sensitize response to Ara-C. In the metagenomic result, we found that several bacteria which were identified as probiotics, such as *L. Acidophilus*, *B. bifidum* and *L. reuteri* were enriched by Curcumin. These bacteria protected intestine through inhibiting inflammation. In contrast, some pathogenic bacteria were decreased, including *E.Coli, A.Muciniphila* and *B.fragilis. E.Coli* and *B.fragilis* were demonstrated to cooperate in invading into deep layer of intestine [22]. *A.Muciniphila* was proved to degrade intestinal mucus to break its intact [23]. In intestine related disease, the break of intestinal mucus was considered to be the initial step of disease, allowing the invasion of bacteria to cause inflammation. Intestinal inflammation allows the dysregulation of TJPs, allowing the intestinal pathogen to invade into deep place of intestine [18]. Here, we also found that Curcumin treatment led to ameliorated TJPs dysregulation, which may be associated with the enriched probiotics. Recently, a study revealed that strengthening intestinal barrier is beneficial for AML chemotherapy through reduction of bacterial translocation [17]. On the other hand, study reported that substantial bacteria was also detected in other cancerous tissues [24], indicating intestinal bacteria can transfer from intestine to other tissues. In our study, we also found that strengthening intestinal intact sensitized response to Ara-C, which was associated with suppressed SQLE induced by Curcumin-mediated microbiota alteration. However, we did not uncover how SQLE was suppressed. Based on the current findings, the possibility of Curcumin-mediated sensitized response to Ara-C is: Curcumin resulted in strengthening intestinal intact, leading to reduced transfer of bacteria (or their metabolites) to the blood, causing SQLE inhibition. Moreover, a positive correlation between intestinal intact breaking bacteria *E.Coli and A.Muciniphila* and cholesterol in MNCs further indicating that up-regulation of cholesterol is associated with intestinal break. As we all know, SQLE is a key enzyme in cholesterol biosynthesis, which has been reported to be oncogenic in several disease [25, 26]. Our study fulfills a further recognition of SQLE in AML.

In conclusion, we demonstrated that Curcumin sensitizes response to Ara-C by regulating microbiota and strengthening intestinal intact is promising in chemoresistant therapy. Moreover, controlling intracellular cholesterol level may be an effective way to ameliorate chemoresistance.

## Supplementary Information

Below is the link to the electronic supplementary material.

Supplementary file1 (TIF 437 KB)

Supplementary file2 (DOCX 13 KB)

Supplementary file3 (DOCX 13 KB)

## Data Availability

The datasets generated during and/or analyzed during the current study are not publicly available due to the on-going related projects but are available from the corresponding author on reasonable request.

## Code availability

N/A.
