# Supplementary figures and images for: Curcumin sensitizes response to cytarabine in acute myeloid leukemia by regulating intestinal microbiota
Source: Cancer Chemother Pharmacol. 2022 Jan 23;89(2):243–53. doi: 10.1007/s00280-021-04385-0 (PMC8807457; doi:10.1007/s00280-021-04385-0)

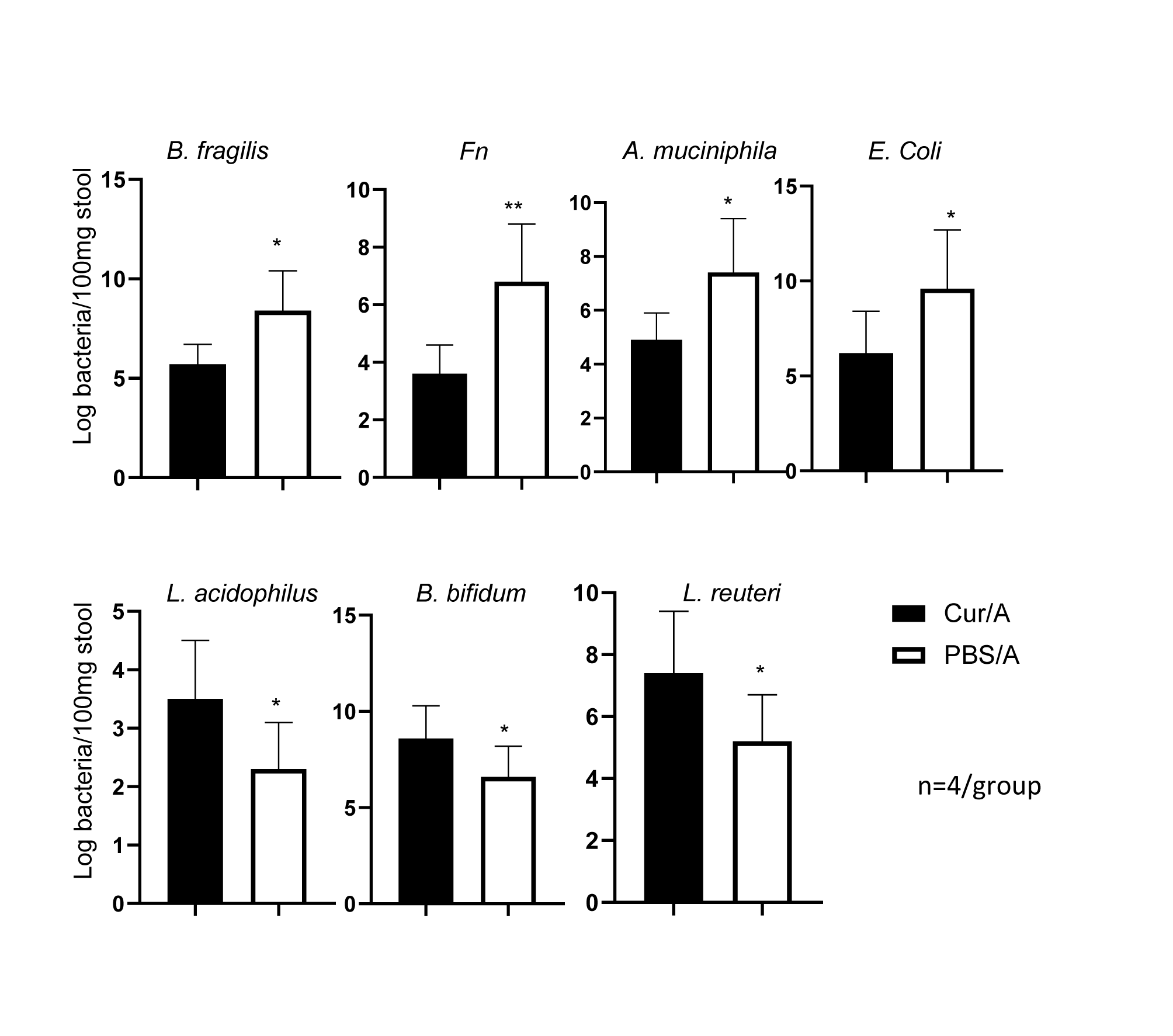

Supplement: Supplementary file 1 — Supplementary file1 (TIF 437 KB) [file 280_2021_4385_MOESM1_ESM.tif]
