# Supplementary material for: Curcumin sensitizes response to cytarabine in acute myeloid leukemia by regulating intestinal microbiota
Source: Cancer Chemother Pharmacol. 2022 Jan 23;89(2):243–53. doi: 10.1007/s00280-021-04385-0 (PMC8807457; doi:10.1007/s00280-021-04385-0)
Supplement: Supplementary file 2 — Supplementary file2 (DOCX 13 KB) [file 280_2021_4385_MOESM2_ESM.docx]

**Figure S1** To Further confirm the synergistic effect between Curcumin and Ara-C, Curcumin (0-100μM) were co-treated to HL-60 and THP-1 cells with Ara-C (3μM). (A-D) Apoptosis and proliferation assay showed that Curcumin did not have a direct synergistic effect on AML cells.

**Figure S2** To address the colonization of bacteria, several Curcumin induced significantly altered bacteria were examined by qPCR. It showed that Curcumin induced enrichment of probiotics were also increased in Cur/A mice stool treated GF mice in concomitant with decreased pathogenic bacteria.

**Table S1** Significantly altered bacteria of species level with Curcumin treatment

**Table S2** Significantly altered metabolites in MNCs of AML mice with Curcumin treatment
